# Study on Type IV Cracking Behavior of T92/Super304H Dissimilar Steel Welded Joints in Long-Term Service

**DOI:** 10.3390/ma17235888

**Published:** 2024-12-01

**Authors:** Denghui Wang, Fei Gao, Zihao Shen, Yuan Li, Zhen Zheng, Mingping Zhou, Fulai Yang, Shichao Zhang, Zheng Zhang

**Affiliations:** 1 School of Materials Science and Engineering, Beihang University, Beijing 100191, China; wangdh@buaa.edu.cn (D.W.); gaofei520@buaa.edu.cn (F.G.); 13583617829@163.com (Z.S.); 18811327152@163.com (Y.L.); zhengzhen@buaa.edu.cn (Z.Z.); zhoump@buaa.edu.cn (M.Z.); buaayangfulai@163.com (F.Y.); zhangshichaomail@163.com (S.Z.); 2 AECC Beijing Institute of Aeronautical Materials, Beijing 100095, China

**Keywords:** heterogeneous steel welded joints, creep failure, type IV cracking, cavities

## Abstract

The microstructure and residual mechanical properties of several groups of T92/Super304H dissimilar steel welded joints (hereinafter referred to as welded joints) in service for 70,000~85,000 h were analyzed. The results show that the early service history of the welded joint results in the polygonization of the martensite lath and the coarsening of the precipitated phase on the side of T92 steel. In the further creep process, the cavities nucleate along the precipitated phase interface and the triple junction grain boundary. Under the same load, the creep life of the joint decreases rapidly with the increase in service time and, finally, type IV cracking occurs. Type IV cracking needs to meet two conditions: 1. a large degree of precipitated phase coarsening and cavity nucleation in the fine grain heat-affected zone (FGHAZ) and 2. a much lower loading stress than the yield strength.

## 1. Introduction

To solve the problem of energy shortages and environmental pollution, thermal power-generating units are developing towards generating higher temperatures and higher pressures. At present, units with higher operating parameters such as supercritical and ultra-supercritical units are recognized as the most feasible and reliable power generation technology under this idea. Due to the increase in temperature and pressure of the unit, the traditional heat-resistant steel materials have been unable to meet the supercritical and ultra-supercritical units. New martensitic/austenitic heat-resistant steels such as T/P92 steel and Super304H steel have become some of the main unit materials [1,2].

Due to the requirements of construction costs and working environments, there are many dissimilar steel welded joints in the unit [3], such as welded joints of T/P92 with Super304H steel, T92/HR3C steel, and P91/SS316L steel [4,5]. The evolution of complex microstructures and properties in harsh environments has been reported in many studies [6,7]. Guohong Chen et al. [8,9] studied the evolution of the microstructure and mechanical properties of T92/Super304H dissimilar steel joints after high-temperature aging. The results show that with the increase in aging time, in the heat-affected zone (HAZ) and base metal (BM) on the T92 side, the precipitates accumulate and coarsen along the prior austenite grain boundary or prior austenite grain. In the HAZ and BM of the Super304H side, the growth of austenite grain and precipitated phase is small, and the fracture position of the aged joint is always in T92 BM. Myung-Yeon Kim et al. [10] studied the creep deformation behavior of the cross weld of T92 and Super304H dissimilar steel welded joints. The results show that the fracture position of the joint is always concentrated on the base metal and heat-affected zone on the side of T92 at 65 °C, and its failure is related to the precipitation of the Laves phase. Up to now, the reason for the failure of dissimilar steel joints is mostly considered to come from the coarsening of precipitated phases.

With the use of dissimilar steel joints in power plants, many joints have been found to fail prematurely or decrease in strength. Experiments show that the microstructure evolution and damage behavior of dissimilar steel joints during long-term use are significantly different from those during short-term creep. Take type IV cracking as an example. Type IV cracking is a common failure mode of power plant joints, but this fracture behavior is difficult to reproduce in the laboratory, so it is difficult to conduct more in-depth research. The failure mode of type IV cracking is related to the long-term evolution of the microstructure of the joint. In the past, the failure behavior and microstructure damage behavior of joints were always studied by preparing joints and conducting short-term creep tests. Few people have studied long-term service materials, joint failure, and microstructure damage. Therefore, in this study, the welded joints of T92/Super304H dissimilar steel which have been in service for 80,000 h are taken as the research object. The damage behavior during long-term service is evaluated, and the difference between long-term service and short-term tests in the laboratory is revealed. At the same time, the type IV cracking behavior is studied and discussed to reveal the micro-mechanism of type IV failure of joints. It provides a direction for further exploring the mechanism of type IV failure and provides a reference for pipeline safety evaluation and failure prevention in engineering practice.

## 2. Experimental Details

### 2.1. Materials

**Figure 1:**
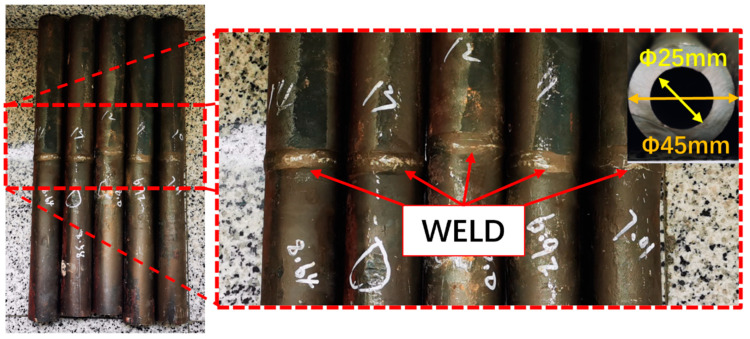
Pipeline materials to be studied.

T92/Super304H dissimilar steel pipes that served 70,000 h, 80,000 h, and 85,000 h in the power plant were used in the study. As shown in Figure 1, the outer diameter of the pipe is 45 mm, the inner diameter is 25 mm, and the wall thickness is 10 mm. In the welded joint, the welding material adopts ENCrNi-3-type welding wire. The service environment of the pipeline is 650 °C.

### 2.2. Test Methods

**Figure 2:**
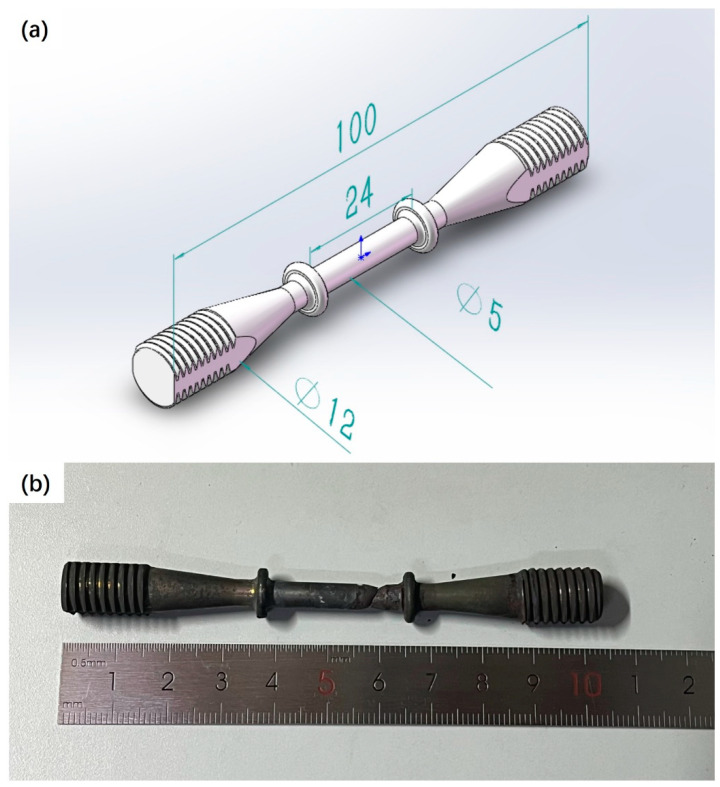
Schematic diagram of creep specimen (**a**) and fracture morphology of the specimen (**b**).

The hardness of the welded joint was measured by a Yinuo FALCON511 microhardness tester, and the micro Vickers hardness was measured according to the standard GBT 4340.1-2009 [11]. The test load was 500 gf and the holding time was 15 s. The surface hardness of the long-term service joint was measured by a matrix method with a horizontal and vertical spacing of 0.5 mm. Before the creep test, the yield strength and tensile strength of welded joints were measured by a Z250 electronic universal material testing machine. The tensile sample was taken from along the axial direction of the pipeline, that is, the tensile direction of the sample was perpendicular to the circumferential weld seam. The tensile rate was 0.00025 s^−1^, and the test temperature was 23 °C and 650 °C. The measured yield strengths at room temperature and high temperature were 418 MPa and 239 MPa, respectively. The creep test was carried out on an RDJ-30 mechanical high-temperature creep testing machine. The size of the creep specimen was determined according to GB/T 2039-2012 [12], as shown in Figure 2, with a total length of 100 mm; the dimension of the gauge length segment was 24 mm × Φ5 mm, and there was a joint located at the center of the gauge length. The specimen was clamped by threads on both sides, and the outer diameter of the clamping end thread was 12 mm. The test temperature was 650 °C, and the test stress was 30% of the yield strength, that is, 70~75 MPa. Figure 2b shows the morphology of the specimen after creep fracture, with the fracture location consistent with the contour of the heat-affected zone in the joint.

### 2.3. Microstructure Characterization

The creep fracture surface was characterized by JSM scanning electron microscopy (SEM). For the fracture with oxidation, a NaOH + KMnO_4_ + H_2_O water bath was used to boil for 120 min, a saturated oxalic acid solution was used for ultrasonic cleaning for 8 min, an acetone solution was used for ultrasonic cleaning for 8 min, a tape + brush were used to repeatedly paste and brush to remove the oxidized structure. The microstructure was polished by 200 #~2000 # sandpaper and mechanically polished. The polished metal was corroded by a ferric chloride hydrochloride solution, and the microstructure was characterized and analyzed by Japanese scanning electron microscope JSM6010 (JEOL Ltd., Akishima, Tokyo, Japan). The device model used for EBSD analysis was Japanese scanning electron microscope JSM-7900 F (JEOL Ltd., Akishima, Tokyo, Japan); the equipment used in TEM analysis was Japanese high-throughput electron microscope JEM-2010 (JEOL Ltd., Akishima, Tokyo, Japan).

## 3. Results

### 3.1. Dissimilar Steel Joints After Long-Term Service

**Figure 3:**
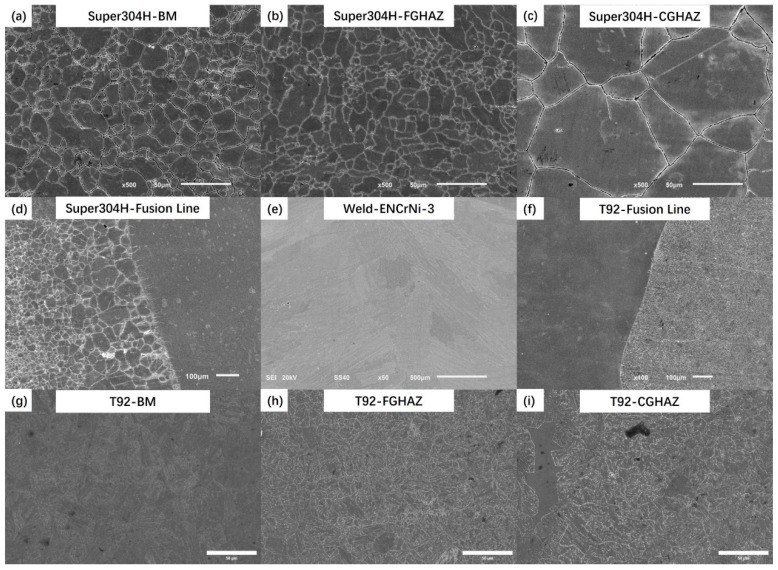
Microstructure of various micro regions in dissimilar steel welded joints.

The welded joint had been in service for more than 80,000 h in the power plant before the test, and its microstructure and mechanical properties had been degraded. Shown in Figure 3 is the SEM image of each micro-zone of the joint, where Figure 3a–d is the side of Super304H steel, Figure 3e is the weld nugget zone (WZ), and Figure 3f–i is the side of T92 steel. The microstructure of the HAZ and the BM on the side of the Super304H steel in the joint is austenite, and there are no large-sized precipitated phase particles in the microstructure. The austenite structure is complete, and the grain boundary characteristics are clear. At the same time, the coarse grain heat-affected zone (CGHAZ) and the FGHAZ maintain their grain size characteristics after welding: The CGHAZ grains are coarse and the grain size of the FGHAZ is fine; its excessive characteristics are shown in Figure 3d, showing obvious gradient variation characteristics. The grains in the WZ are coarse, which is much higher than other areas of the joint. The microstructure on the side of T92 steel is a martensite lath structure. Many white precipitated phases can be seen in the martensite lath structure and distributed along the interface. A small number of grains without precipitated phases can be observed in the HAZ of T29 steel, which is δ-ferrite. Compared with the side of Super304H steel, the fusion line on the T92 side is clearer.

**Figure 4:**
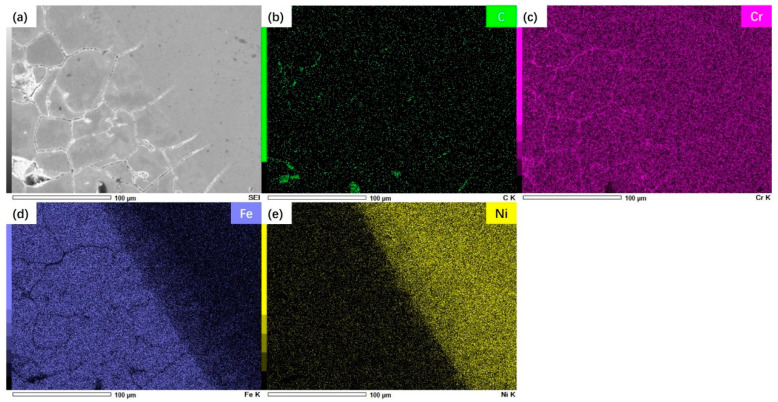
EDS analysis results of Super304H steel side in welded joints: (**a**) Microstructure morphology; (**b**) C element distribution; (**c**) Cr element distribution; (**d**) Fe element distribution; (**e**) Ni element distribution.

**Figure 5:**
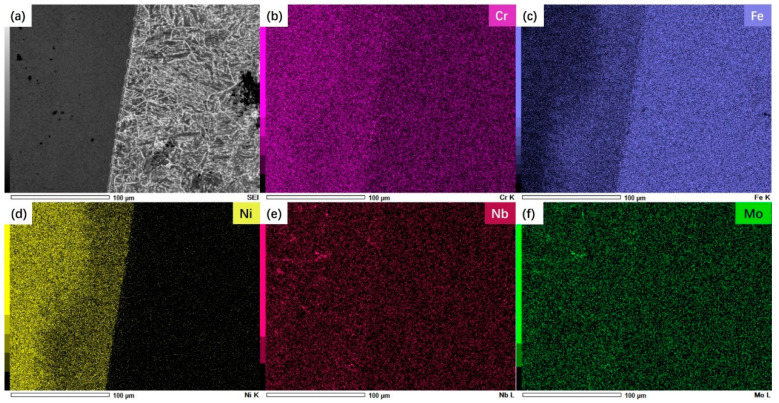
EDS analysis results of T92 steel side in welded joints: (**a**) Microstructure morphology; (**b**) Cr element distribution; (**c**) Fe element distribution; (**d**) Ni element distribution; (**e**) Nb element distribution; (**f**) Mo element distribution.

For welded joints, there are some differences in the chemical composition between the WZ and the materials on both sides. Therefore, in the long-term high-temperature service process, the chemical composition will have obvious diffusion phenomena. At the same time, there are also obvious differences in the element distribution characteristics on both sides of the WZ, as shown in Figure 4 and Figure 5.

Figure 4 is the Super304H steel side of the joint. It can be seen from the figure that the main element difference between the Super304H steel and the WZ is that the content of Fe on the Super304H side is high, while the content of Ni in the WZ is higher. The large concentration gradient promotes the diffusion of Fe to the WZ under the long-term high temperature environment, resulting in the dilution of the Ni element at the edge of the WZ. Therefore, the segregation of C and Cr can be seen at the grain boundary of CGHAZ in Super304 H steel, which is related to the aggregation of precipitates at the grain boundary.

Figure 5 shows the chemical composition distribution of the T92 side of the weld. The Fe diffuses from the T92 steel side to the WZ, forming a diffusion layer nearly 70 μm wide. The diffusion of Fe causes the content of the Ni element in this area to decrease, while the diffusion of the Fe element does not seem to affect the concentration of Cr, Nb, Mo, and other elements. Different from the Super304H side, the elements on the T92 side do not form obvious segregation, and the element distribution is more uniform.

**Figure 6:**
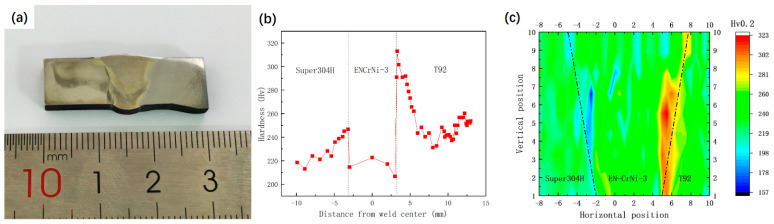
Hardness distribution before and after joint service: (**a**) macroscopic morphology of joint cross-section; (**b**) hardness distribution before service; (**c**) hardness distribution after 80,000 h of service.

From the EDS results, obvious element diffusion occurred in the WZ during long-term use, while the matrix structure on both sides of the WZ remained relatively stable. Figure 6 shows the hardness distribution of the joints before and after use. The overall width of the joint is within 10 mm. Among them, Figure 6b shows the hardness distribution of the unserved joint. It can be seen from the figure that the CGHAZ on the side of T92 steel in the joint has the highest hardness, and the maximum hardness is 315 Hv, while the hardness of FGHAZ on this side is the lowest at about 240 Hv. The hardness of Super304H steel is lower than that of T92 steel and higher than that of WZ, the hardness in this area decreases from the WZ to BM, and the hardness is in the range of 215~245 Hv. The WZ has the lowest hardness in the joint, with an average of 220 Hv. The hardness distribution of the joint after 80,000 h of service is shown in Figure 6c. The hardness of the BM and WZ on both sides of T92 and Super304H is consistent, about 220 Hv, that is, the hardness of BM on both sides of T92 and Super304H decreases, while the hardness of WZ remains unchanged. In addition, the CGHAZ on the side of T92 steel is still the region with the highest hardness in the joint, and its maximum value is about 310 HV.

**Figure 7:**
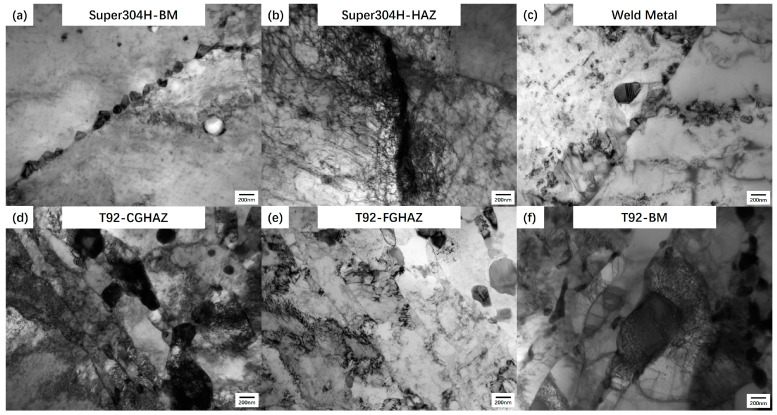
TEM microstructure of welded joints after 80,000 h of service.

The TEM analysis of welded joints with 80,000 h of service was carried out, and the characteristics are shown in Figure 7. In the BM and HAZ of the austenitic steel side, the precipitated phases are mainly an MX phase with chain distribution along the grain boundary and a Cu-rich phase with random distribution in the grains. The diameter of the MX phase is about 200 nm, while the diameter of the Cu-rich phase is about 50 nm. The grains in the WZ are coarse, the precipitated phases are rare, there is not a large number of dislocations in the grains, and the structure is relatively stable. The characteristics of tempered martensite can be observed in the BM and HAZ on the side of T92 steel. In addition, the coarsening degree of the precipitated phase in the BM is low, while the phase in the middle of the HAZ is obviously coarsened, and its diameter can reach more than 300 nm.

### 3.2. Creep Fracture Behavior

**Figure 8:**
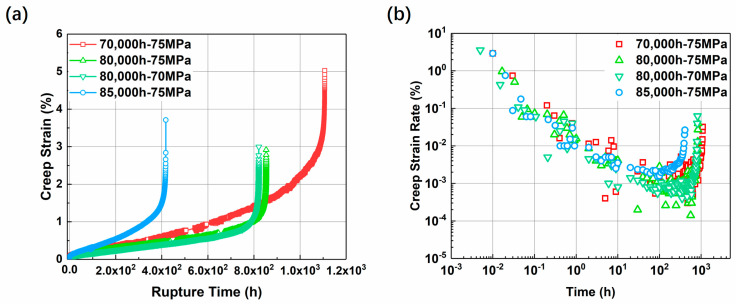
Creep curves (**a**) and creep strain rate curves (**b**) of welded joints with different service histories.

By analyzing the welded joints after service, it can be seen that the microstructure and properties of dissimilar steels have deteriorated under long-term high temperature and stress. The creep performance degradation of dissimilar steel joints with a service history of 70,000 h to 85,000 h is shown throughout the creep test in Figure 8. From the figure, it can be seen that the longer the service history, the shorter the creep life. Under the same temperature and stress, the joint with the longest service time of 85,000 h has a higher creep strain rate, which leads to more plastic strain accumulation and earlier fracture failure, followed by the joint with 80,000 h. The creep life of the joint with 70,000 h is the longest and the strength is relatively the best.

It is worth noting that although the variable strain rate of the joint serving for 70,000 h is higher than that of the joint serving for 80,000 h, its plastic deformation ability is significantly better than that of the joint serving for 80,000 h. During the service process of the joint, the loss of creep performance is not only reflected in the decrease in creep life under the same stress, but also in the decrease in plastic deformation ability with the deterioration of the structure.

**Figure 9:**
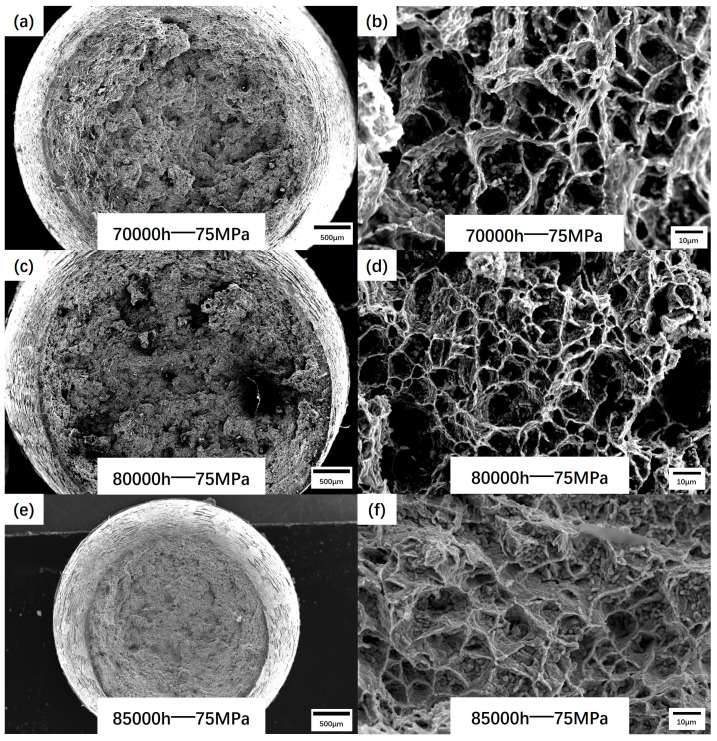
Creep fracture surface: (**a**,**b**) are the creep fracture surfaces of the 70,000 h group joint; (**c**,**d**) are the creep fracture surfaces of the 80,000 h group joint; (**e**,**f**) are the creep fractures of the 85,000 h group joint.

Different from the un-serviced welded joints, the fracture mode and fracture position of the specimens have changed greatly due to further creep, as shown in Figure 9. The fracture position of the welded joint in the un-serviced state mostly occurs in the BM on the T92 side. When the fracture occurs, a certain necking occurs on the fracture surface, showing the characteristics of ductile cracking. When the joint is broken after long-term service, the position of the creep fracture is concentrated in the FGHAZ of the T92 steel, and the fracture has no obvious necking. Macroscopically, it is brittle, while microscopically, it can be observed that it is still dominated by dimples. From the comparison of the depth and diameter of the dimples, the longer the service time of the joint, the weaker the deformation ability on the microscopic scale.

### 3.3. Microstructural Evolution

**Figure 10:**
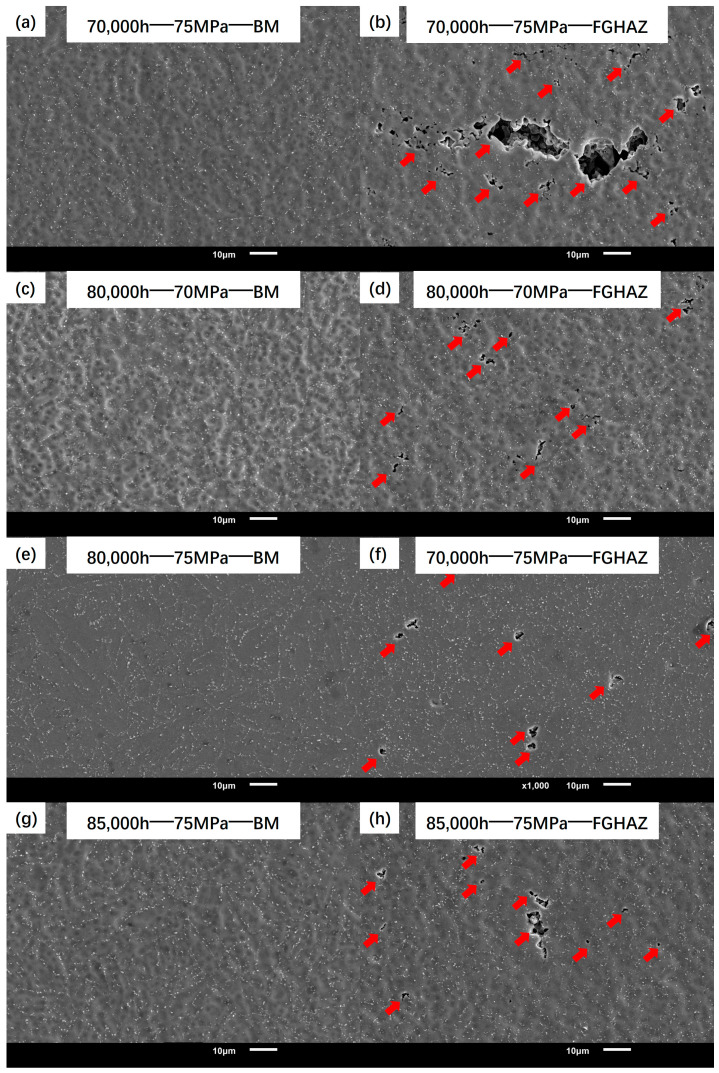
Sectional microstructure of various subzones in creep specimens.

Figure 10 shows the microstructure of the creep fracture section. It can be seen that there is a lot of creep damage in the FGHAZ on the T92 side of the joint. The damage is manifested as discrete creep cavities or discontinuous microcracks, as indicated by the red arrows in the figure. Comparing different microstructures in three joints, it can be found that the cavities in the FGHAZ of 80,000 h and 85,000 h, with poor toughness, maintain a small size, and the dispersed cavities are mostly wedge-shaped or round [13]. In contrast, the toughness of the 70,000 h joint is higher than that of the other two specimens. Some adjacent cavities in the FGHAZ merged to form cracks.

It is not difficult to see that the cavity nucleation and growth is only concentrated in the FGHAZ of T92 steel. Figure 10a,c,e,g show the microstructure of the BM on the T92 side of the fracture in each group, and no cavity characteristics are found. The characteristics of many cavities shown in the FGHAZ are also not observed in other subzones of the joint.

**Figure 11:**
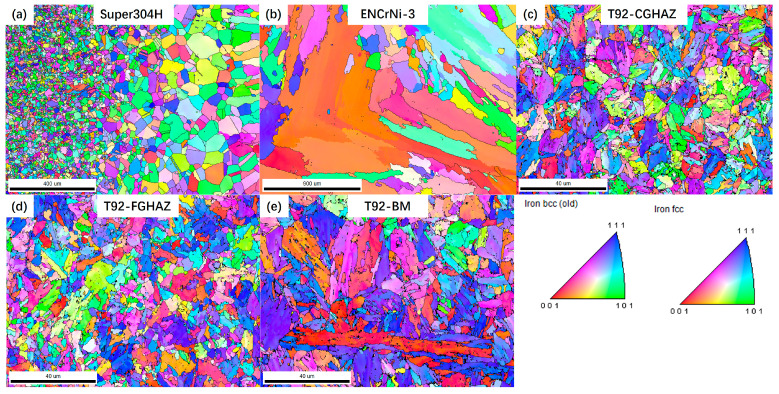
Microstructure of various subzones in welded joint with service life of 80,000 h: (**a**) BM on Super304 side; (**b**) WZ; (**c**) CGHAZ on T92 steel; (**d**) FGHAZ on T92 steel; (**e**) BM on T92 side.

The cavity distribution has obvious regional characteristics, which is directly related to the microstructure evolution of each subzone. Figure 11 shows the inverse pole figure (IPF) of each subzone of the joint after 80,000 h of service. The figure shows the grain size characteristics and orientation characteristics of each subzone. Overall, each micro-zone of the joint maintains the grain size characteristics formed during welding, and the grain orientation does not deflect in a specific direction due to temperature or stress. It is worth noting that in the FGHAZ on the T92 steel side, the average grain size is small, and there are few martensitic laths inside the grains.

The KAM method is a method to determine the geometrically necessary dislocation (GND) density based on the orientation difference angle between any two adjacent points and the distance between the two points [14,15,16,17]. According to the strain gradient model [18], the relationship between the GND density and the orientation angle can be expressed as follows:

(1) $$∆\theta_{i}=\frac{\sum_{j=1}^{n}\left|\theta_{j}^{sur}-\theta_{i}\right|}{n}$$

(2) $$\rho_{GND}=\frac{2∆\theta_{i}}{\mu b}$$

**Figure 12:**
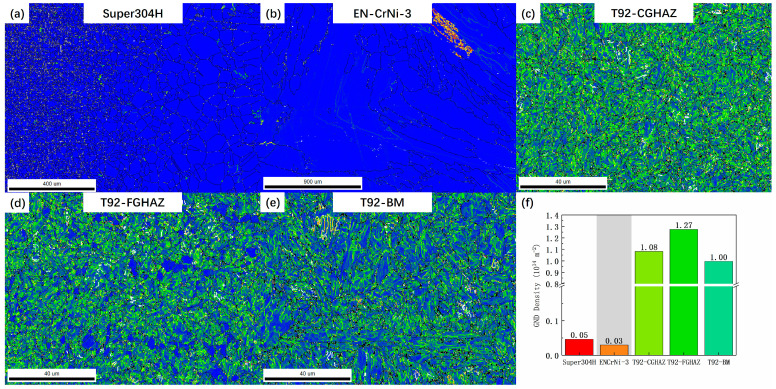
GND distribution of different subzones of welded joints in service for 80,000 h: (**a**) Super304H; (**b**) EN-CrNi-3; (**c**) CGHAZ on the T92 steel side; (**d**) FGHAZ on the T92 steel side; (**e**) BM on the T92 steel side; (**f**) The average GND density in each subzone of the joint.

**Figure 13:**
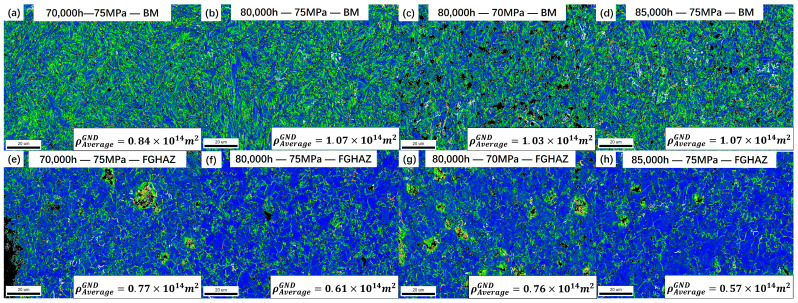
Distribution characteristics of GND after creep fracture.

In the formula, ${∆\theta }_{i}$ is the local misorientation angle of the corresponding point *i*, $\theta_{j}^{sur}$ is the misorientation angle of the adjacent point *j*, $\rho_{GND}$ represents the GND density, and *μ* is the unit length. In this case, *μ* is 200 nm and 2000 nm, respectively, and *b* is the Burgers vector. In this work, the misorientation angle exceeding 2° has been excluded, as shown in Figure 12 and Figure 13.

Figure 12 shows the distribution of geometrically necessary dislocations in each subregion of the welded joint after 80,000 h of use. The relevant data were characterized by the EBSD method and calculated using OIM Analysis 7 software. Currently, the dislocation density in the Super304H side and the WZ of the joint is the lowest, and there is almost no distribution of geometrically necessary dislocations. The T92 steel side has a higher degree of GND density, and the dislocation density in the fine grain heat-affected zone on the T92 steel side is the highest, about 1.27 × 10^14^ m^−2^, followed by the CGHAZ and the BM. The distribution of GND in the joints with 70,000 h and 85,000 h of service is consistent with this.

The GND density of the welded joints after the creep test was analyzed. As shown in Figure 13, it can be seen that after creep fracture, the GND density in the FGHAZ decreased significantly, and the dislocation density was only about 50% of that before creep. In other subzones of the joint, the change in the dislocation density was very small. For example, the decrease in dislocation density in the BM was about 15%, which is much lower than that in the FGHAZ. It is worth mentioning that the dislocations in the FGHAZ only had a high degree of aggregation near the cavities, while there were almost no new dislocations generated or retained inside the grains.

**Figure 14:**
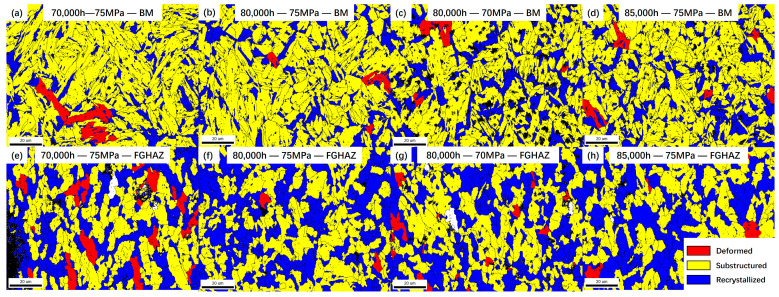
GOS distribution in various subzones of creep fracture specimens.

**Figure 15:**
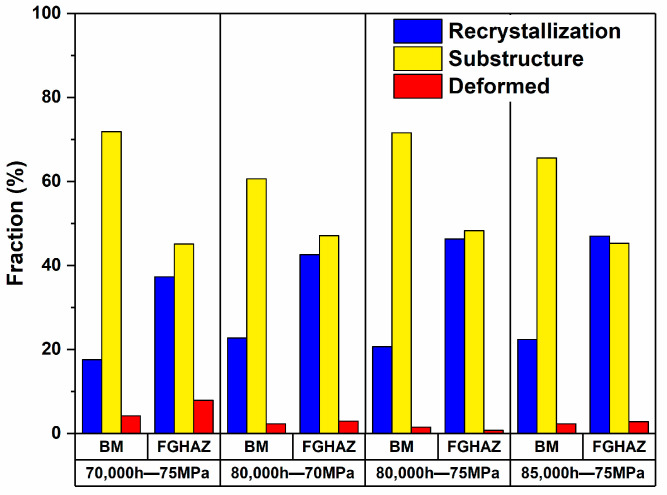
Statistical analysis of the proportion of BM and FGHAZ grains in creep specimens.

The dislocation density of the FGHAZ on the T92 side of the welded joint decreased, which is related to the recrystallization of the crystal. The grain orientation distribution diagram (GOS) can distinguish the recrystallized grains, substructure grains, and deformed grains. In Figure 14, the grain types of the BM and the FGHAZ in the four groups of specimens are analyzed. It can be seen in the figure that a large number of recrystallized grains can be observed in the FGHAZ of the four groups of specimens. The proportion of recrystallized grains is significantly higher than that of the BM of the same specimen. Taking the FGHAZ and the BM with 80,000 h of service as an example, the proportion of recrystallized grains in the BM is about 20.7%, while the proportion of recrystallized grains in the FGHAZ is 46.3%, which is more than doubled. The proportion of deformed grains is about 1%, as shown in Figure 15.

**Figure 16:**
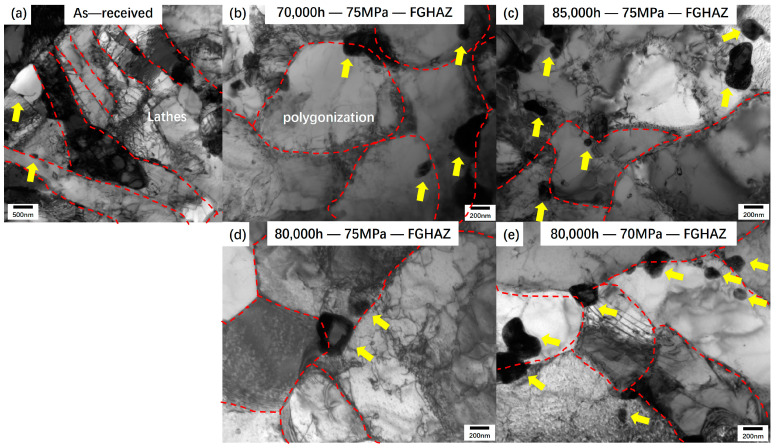
Microstructure characteristics at the fracture surface of creep fracture specimens.

The microscopic characteristics near the fracture of the joint samples under three working conditions are shown in Figure 16. Figure 16a shows the microscopic characteristics of samples with no service history. After welding heat treatment, the characteristics of martensite laths can be observed, as shown by the red dotted line, and the laths show a slender parallel distribution. There is a dislocation network in the lath, and there is a distribution of precipitated phases at the grain boundaries. After the creep test, the martensite lath characteristics disappeared and many subgrain structures were formed. As shown in the red dotted line in Figure 16b–e, the average size of the precipitated phase increased. As shown by the yellow arrow, the size of the precipitated phase before service was less than 100 nm, while the diameter of the precipitated phase in the microstructure after creep fracture was generally greater than 200 nm, and some of the particle diameters were even greater than 300 nm. It is worth noting that under the condition of 75 MPa, the longer the use time, the larger the subgrain diameter. It is worth noting that in the microstructure after creep fracture, the number of dislocation lines in the subgrains is significantly reduced, and only a small amount of dislocation tangles can be observed at the grain boundaries and subgrain boundaries. The order of subgrain diameter of the three groups of samples is 70,000 h > 80,000 h > 85,000 h, corresponding to the creep time of the three groups of samples in Figure 8.

## 4. Discussion

The hardness and tensile properties of T92/Super304 H dissimilar steel welded joints decreased significantly after 70,000~85,000 h of service in the power plant. For dissimilar steel welded joints, the decrease in joint strength is related to the change in joint microstructure and composition. Figure 3 reflects the microstructure characteristics of each subzone of the joint. Compared with the microstructure before service, the grain characteristics of the austenite steel side did not change significantly, while the martensite steel side experienced relatively obvious degradation, and its grain boundary profile and martensite lath pattern gradually blurred. In addition, the number and size of precipitated phases in the HAZ on the side of the martensite steel also increased significantly. The thermal stability of the martensite structure on the side of the T92 steel was worse than that of the austenite structure on the side of the Super304H steel, which was the fracture position during creep. The long-term high-temperature environment also promoted the diffusion of chemical elements in each region of the joint, as shown in Figure 4 and Figure 5. The segregation of the Cr element at the grain boundary will promote the coarsening of the precipitated phase at the grain boundary, on the one hand, and, on the other hand, it will also promote the occurrence of intergranular corrosion, resulting in a decrease in intergranular strength.

The creep test of the welded joints in service found that the creep life of the welded joints with different service histories also decreased significantly under the same stress. As shown in Figure 8, the creep life of the welded joint specimen in service for 70,000 h is about 1100 h when creeping at 650 °C—75 MPa, the creep life of the joint specimen in service for 80,000 h is reduced to about 800 h, and the service time of the joint specimen in service for 85,000 h is only 400 h. It can be seen that the increase in service time does not linearly reduce the residual creep life under the same conditions. For pressure equipment, such a rate of life attenuation is very dangerous.

For the welded joints that have been in service, the pre-service environment is a kind of high-temperature and low-stress environment. During the long-term service process, the changes in the microstructure are mainly manifested in the deterioration of grain boundary characteristics and the precipitation and coarsening of new precipitated phases. No defects such as cavities and cracks were observed in the early stage. After creeping under higher stress, type IV cracking occurs in the joint, that is, the fracture position is concentrated in the FGHAZ. A large number of cavities were observed near the fracture after creep fracture. This feature only existed in the FGHAZ on the side of the joint T92 steel. The cavities showed circular or wedge-shaped characteristics, and their sizes were mostly below 5 μm. Some adjacent cavities gradually merged under stress to form microcracks. Adjacent microcracks further merged to form main cracks, resulting in overload fracture when the local net cross-sectional area was too small.

When the welded joint is serviced in a high-temperature environment, the high temperature and low stress will promote a slow change in the microstructure of each subzone of the joint. In contrast, the grain size of the FGHAZ on the martensite side with worse thermal stability is small, the proportion of grain boundaries is relatively high, and the number and size of undissolved precipitates are high, which will promote the coarsening of the precipitates in this area. The degree is higher and the cavities are easier to nucleate here. If the service stress is constant, the FGHAZ on the martensite side will undergo a longer evolution, forming a higher number of cavities and resulting in final failure; the sudden increase in stress will promote the nucleation of cavities in the area and prematurely cause local overload, resulting in joint failure. The occurrence of type IV cracking is related to the coarsening of precipitated phases and the nucleation of cavities during long-term service. The coarsening of precipitated phases and the nucleation of cavities in the microstructure are the necessary conditions for type IV cracking, and a yield stress far below 1/2 is the sufficient condition for type IV cracking. When both are satisfied at the same time, type IV cracking will occur.

## 5. Conclusions

In this study, the creep fracture behavior of T92/Super304H dissimilar steel welded joints with more than 70,000, 80,000, and 85,000 h of service in power plants at 650 °C was studied. The creep fracture behavior and failure mechanism of T92/Super304H dissimilar steel welded joints were studied. The following conclusions are drawn:

1. The microstructure and mechanical properties of T92/Super304H dissimilar steel welded joints after long-term service have changed, and there is a large degree of element diffusion in the joints, among which the mutual diffusion degree of the Fe element and Ni element on the Super304H side is the highest. The diffusion degree of elements on the T92 side is low. The tensile strength of the joint at room temperature does not decrease significantly, but the tensile strength at high temperature decreases seriously. The tensile strength of the joint at 650 °C was about 254 MPa, which is 13% lower than that before service;
2. The failure location of austenitic/martensitic dissimilar steel welded joints is mostly concentrated on the martensitic side with poor thermal stability. The coarsening of precipitated phases and the nucleation of cavities on this side are necessary conditions for type IV cracking, and external stress far below half of the yield strength of the joint is a sufficient condition. Only when both factors work together can type IV cracking occur, that is, long-term low-stress creep deformation can easily form type IV cracking in the joint;
3. During the long-term service of dissimilar steel joints, the microstructure is degraded, and the microstructure characteristics of joints serving for 70,000 h, 80,000, h and 85,000 h are basically the same, while the creep performance decreases with the increase in service time;
4. In accelerated experiments under high stress, the grains in the FGHAZ of dissimilar steel joints undergo recrystallization, resulting in the annihilation of a large amount of GND and a significant decrease in GND density, leading to a significant decrease in the strength of the matrix in this area. The high proportion of coarsened precipitates and trident grain boundaries provides nucleation points for the initiation of creep cavities. The rapid increase in the number of cavities and the merging of adjacent cavities into cracks cause a rapid decrease in the effective strength and effective bearing area of this area, ultimately leading to the fracture of the joint in this area, known as type IV cracking.

## Data Availability

The original contributions presented in this study are included in the article. Further inquiries can be directed to the corresponding author.
